# Recent Advances in the Diagnosis and Management of Congenital Heart Disease

**DOI:** 10.3390/children11010084

**Published:** 2024-01-11

**Authors:** P. Syamasundar Rao

**Affiliations:** Children’s Heart Institute, University of Texas at Houston McGovern Medical School, Children’s Memorial Hermann Hospital, 6410 Fannin Street, Suite #425, Houston, TX 77030, USA; p.syamasundar.rao@uth.tmc.edu

Congenital heart defects (CHDs) are structural abnormalities of the heart or blood vessels that occur while cardiac structures are being formed in utero. However, the age at which the CHD manifests varies. The incidence of CHD is roughly 0.8% in live-born babies. Nearly half of these patients may be managed via the administration of drugs, surveillance, and periodic re-evaluation with no need for surgical or transcatheter procedures. Nonetheless, the remaining 50% of children have required surgery in the past. Since the introduction of transcatheter methods, half of these patients (25% of the entire group) may be treated via non-invasive, percutaneous procedures. Diagnostic methods and management techniques for CHD—the early recognition of neonates with severe CHD, rapid transfer of such infants to hospitals providing suitable care, availability of highly reliable diagnostic tests, developments in the care of neonates, provision of safe anesthesia, inventions of percutaneous techniques, and the ability to perform intricate surgery in the newborn babies and infants—have been developed and improved to such an extent that most CHDs can be identified in a timely manner and “corrected”, and the CHDs that cannot be corrected can be effectively palliated. Advances in therapy practices by medical, surgical, and percutaneous means begun in the late 1930s and have persisted until today. The objective of this Special Issue is to collate papers that address contemporary developments in the diagnosis and treatment of CHDs and present this vital information for the benefit of doctors who treat patients with CHD.

In the first paper, I review the management of acyanotic CHDs. Following a brief discussion of the historical aspects of developing treatment for heart defects, both by surgical [1,2,3,4,5,6] and transcatheter interventions [7], I review the indications, timing, and methods of intervention for the most common acyanotic heart defects [8]. For valvar and vascular obstructive lesions, peak-pressure gradients across the obstructive lesion (50 mmHg for pulmonary stenosis and aortic stenosis and 30 mmHg for aortic coarctation) are used for intervention. Balloon valvuloplasty is generally recommended for both pulmonary and aortic stenosis. For aortic coarctation, surgery in newborns and young infants, balloon angioplasty in children, and stent implantation in adults and adolescents seem to be the current trends. For septal defects, the dimensions of the defect and the magnitude of the shunt across the defect are used as indications for intervention. For atrial septal defects, muscular ventricular septal defects (VSDs), and patent ductus arteriosus transcatheter occlusion is preferred, while surgical closure for membranous VSDs and atrioventricular septal defects is indicated. It was concluded that the presently accessible medical, percutaneous, and surgical methods related to acyanotic CHDs are achievable, reliable, and successful.

In the second paper, Dr. Singh from St. Louis, MO, discusses issues related to aortic valve stenosis (AS) in children. He states that AS is a CHD causing a fixed obstruction of the left ventricular outflow tract and that lesion severity increases with time. He reviews pathologic anatomy, pathophysiology, clinical features, techniques used for diagnosis, natural history, and the treatment of AS. While most children with AS are asymptomatic, newborn babies and young infants may progress to congestive heart failure. Dr Singh details percutaneous [9] and surgical [10] procedures to address AS patients. He recommends balloon aortic valvuloplasty (BAV) due to the lower mortality and morbidity seen with BAV. He asserts that many of these patients require reintervention.

In the next paper, Dr. Sinha and her colleagues from Houston, TX, present a systematic review of the value of pulmonary valve Z-scores in predicting valve-sparing repair (VSR) for the tetralogy of Fallot. In the past, several strategies to protect the development of dilatation of the right ventricle (RV) following the surgical correction of tetralogy of Fallot [11] have been advocated. To further investigate these issues, Dr. Sinha et al. performed searches via PubMed, EMBASE, and Scopus, reviewing a total of 712 papers, 15 of which met the set criteria. Tetralogy patients with pulmonary atresia, multiple aorto-pulmonary collateral vessels (MAPCASs), absent pulmonary valve syndrome, and those associated with atria-ventricular septal defects were excluded. A total of 1091 children who met the criteria; their median age was 6.9 months and median weight was 7.2 kg. VSR was undertaken based on an intra-procedural assessment of the pulmonary valve Z-scores in 14 of these 15 investigations. The median pulmonary valve Z-score was −1.7 with a range from 0 to −4.9. During a follow-up period of 2.83 years (range: 1.4 to 15.8 years), the median of repeat intervention rate was 4.7% with a range from 0 to 36.8%. They found no correlation between low Z scores and the need for re-intervention to address RV outflow tract obstruction. They found that pulmonary valve Z scores secured using echocardiography were not predictive of VSR, but intraoperative Z scores following relief of other RV obstructive lesions appear to be better indicators. Nevertheless, the longer follow-up periods are necessary to confirm the described observations.

In the fourth paper, I review the treatment of the most common cyanotic CHDs. There are several variants of Fallot tetralogy, and each of them requires separate methods. Complete surgical correction may be performed for simple tetralogy Fallot (TOF); some babies may need preliminary palliation with a modified Blalock–Taussig (MBT) shunt. Other variants of TOF, such as TOF with pulmonary atresia, TOF with MAPCAs, and TOF with absent pulmonary valve syndrome require different surgical approaches. Infants with transposed great vessels (both with intact ventricular septum and VSD) require a Jatene procedure, whereas babies with both pulmonary stenosis and VSD are treated using a Rastelli procedure. Many of these infants may require an initial administration of prostaglandin E_1_ infusion and/or Rashkind’s atrial septostomy. Babies with a diagnosis of tricuspid atresia need to be palliated first with either an MBT shunt or pulmonary arterial banding, with a plan for staged Fontan with bidirectional Glenn followed by fenestrated Fontan. Newborns with total anomalous pulmonary venous return are treated by connecting the left atrium with the common pulmonary vein. Infra-diaphragmatic forms require emergency surgery, while supra-diaphragmatic types may undergo the procedure electively. Infants with common truncus are treated by placing a valved conduit between the RV and pulmonary artery in concert with closure of the VSD. Babies with hypoplastic left heart syndrome require a Norwood procedure followed by staged Fontan. Other CHDs such as pulmonary atresia with intact ventricular septum, double-outlet right ventricle, double-inlet left ventricle, and univentricular hearts mostly require a staged surgical approach [12]. The present medicinal, percutaneous, and operative methods to treat cyanotic CHD are reliable and efficient and are undertaken with reasonably minimal risk.

In the next paper, Dr. Sexena from New Delhi, India, discusses the status of cardiac care of pediatric patients in developing countries. She asserts that a majority of patients with congenital and rheumatic heart diseases do not have access to adequate medical/surgical care in developing countries. She describes the inadequacies of health care systems to address heart disease burden and suggests some solutions, such as collaboration between governmental and non-governmental institutions, to tackle these problems [13].

In the final paper of this series, Gupta-Malhothra from Saint Petersburg, FL, and her associates, review multimodal imaging. They list the types of noninvasive multimodality cardiac imaging techniques available in pediatric cardiology practice—echo-Doppler studies, magnetic resonance imaging (MRI), computed tomography (CT), and nuclear imaging—discussing the advantages and limitations of each technique. Quality control, how to develop multi-modality imaging facilities, and the importance interface between radiology and pediatric cardiology services were also reviewed. They conclude that the multi-modality imaging techniques are necessary for implementing custom design methods of transcatheter interventions, electrophysiologic procedures, and surgical therapies, as well as stressing the importance of a team approach [14].

While not all advances were reviewed due to space limitations, substantial developments in diagnosing and managing CHD were touched upon. I and the authors of the papers in this Special Issue hope that these reviews are helpful to readers and will benefit doctors in the treatment of their patients.
